# Creating and using a simulation model to practice sub-Tenon's anaesthesia

**Published:** 2025-10-29

**Authors:** Althea le Roux, James Rice

**Affiliations:** 1 Ophthalmic Surgical Skills Trainer: University of Cape Town Community Eye Health Institute, South Africa.; 2 Consultant Ophthalmologist: Division of Ophthalmology, University of Cape Town, South Africa.

**This simple, low-cost model can be used for teaching and practising your skills.**

Most ocular surgery is performed under local anaesthesia. Anaesthetic options include: topical (eye drops), intracameral, sub-Tenon's, peribulbar and – less commonly – retrobulbar injections. Clinicians choose the type of anaesthesia based on patient and ocular factors, the type of surgery, and the expected duration of the procedure. Patient monitoring in case of an adverse event related to anaesthetic administration is a basic requirement.

We present the construction and use of a simple, low-cost simulation model for teaching and practicing sub-Tenon's injections. Trainees learn the relevant anatomical landmarks and details necessary for safe performance of the technique.

Similar training models have been described elsewhere.^1^

## How to make the simulation model

**Figure 1:**
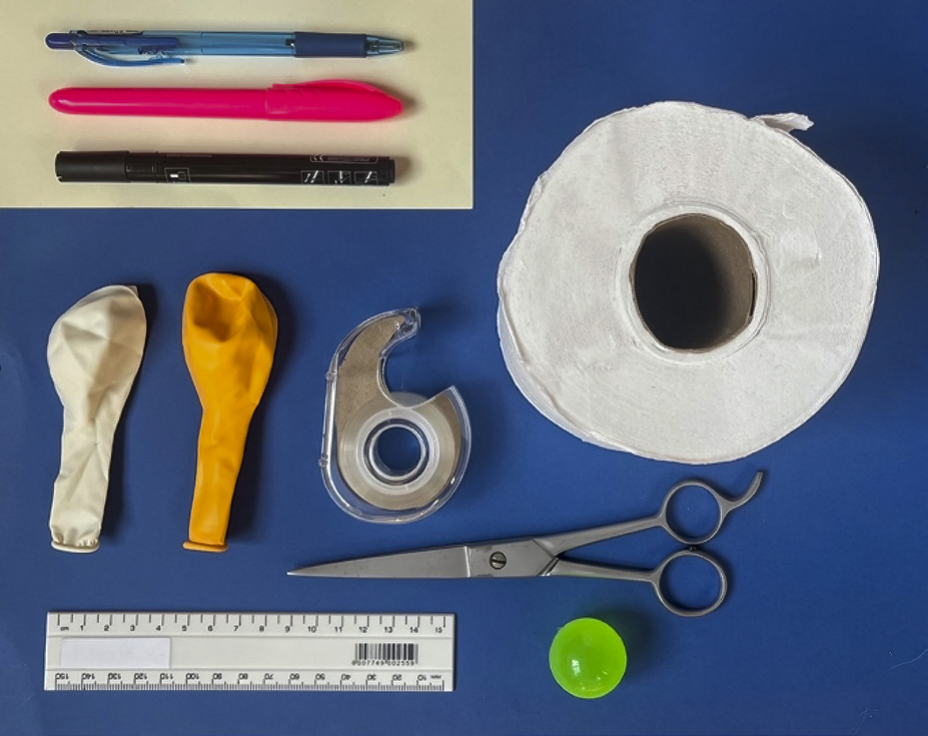
What you need to make the simulation model.

What you will need (Figure 1):

- A rubber ball of approximately 25 mm diameter (a polystyrene ball can also be used)
- 2 small rubber balloons in different colours
- Ballpoint or felt-tip pens in different colours
- Thin cardboard
- Adhesive tape
- Scissors
- A ruler
- A roll of toilet paper

© ALTHEA LE ROUX CC BY-NC-SA 4.0

**Note:** If you are using a polystyrene ball, put some ultrasound jelly or cooking oil on the ball before covering it with the balloons. This will support the gliding action of the instruments.

**Figure 2:**
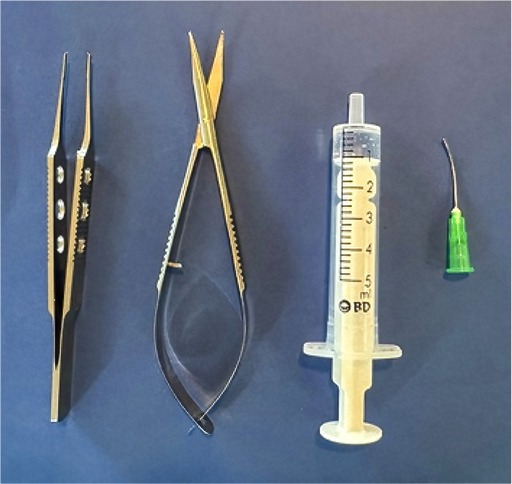
Instruments for the procedure. From left to right: tissue forceps, Westcott scissors, syringe, sub-Tenon's cannula.

Instruments required for the procedure (Figure 2):

- Hoskins #18 tissue forceps or similar
- Blunt-tipped Westcott curved scissors
- 5 ml syringe filled with water
- Sub-Tenon's cannula (the irrigation port of a Simcoe cannula can also be used)

### How to make the model

Push the ball (Figure 3a) into the first balloon. Cut off the remaining part of the balloon (Figure 3b), so that the ball is just covered (Figure 3c).

**Figure:**
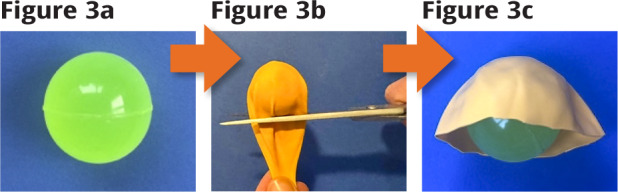


**Figure 4:**
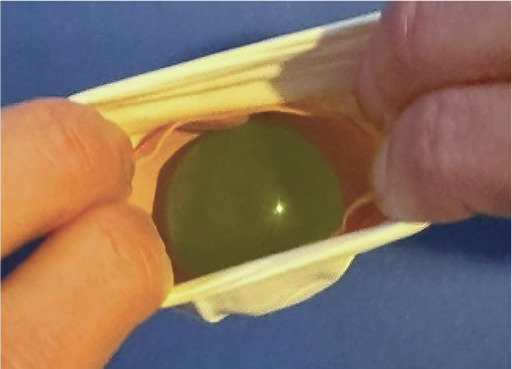


Next, push the (now covered) ball into the second balloon (Figure 4), ensuring that the first balloon remains centred around the ball.

The first layer represents the Tenon's fascia, and the second the conjunctiva.

Cut off the thickened, round edge (or lip) of the second balloon (Figure 5a). This can now be used as an elastic to tie the second balloon at the base of the covered ball (Figure 5b).

**Figure:**
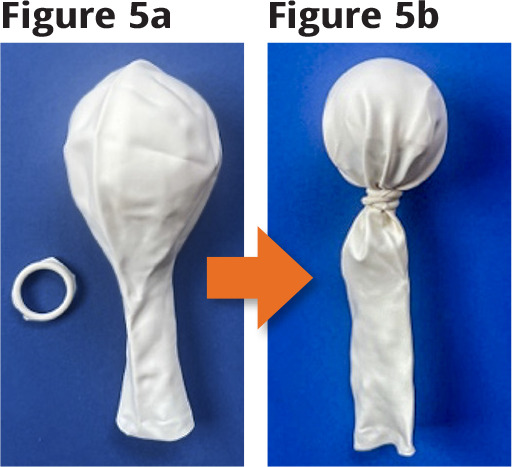


**Note:** The balloons should not be stretched or tight when fitted around the ball; they should be slightly loose.

At the top of the ball, draw a circle with a diameter of approximately 12 mm to represent the corneal limbus. Colour in the iris and pupil (Figure 6a). Next, draw the rectus muscles, starting 5–7 mm from the limbus. This helps with teaching the anatomical landmarks and orientation (Figure 6b).

**Figure 6a:**
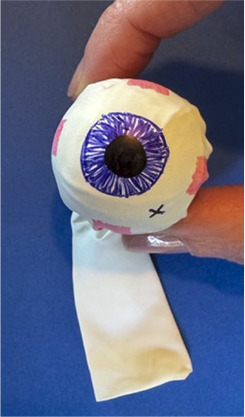


**Figure 6b:**
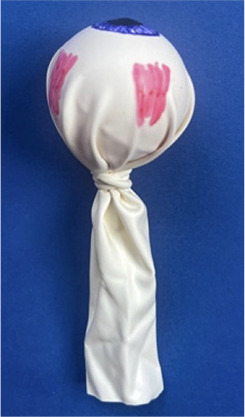


### How to make the cone

Using the thin cardboard, cut a semicircle with a diameter of 12 cm, as shown in Figure 7a. Fashion a small cone as shown in Figure 7b. The cone opening should be slightly wider than the diameter of your toilet roll's inner cardboard cylinder (> 4.5 cm), see Figure 7c. Secure the overlapping parts with adhesive tape.

**Figure:**
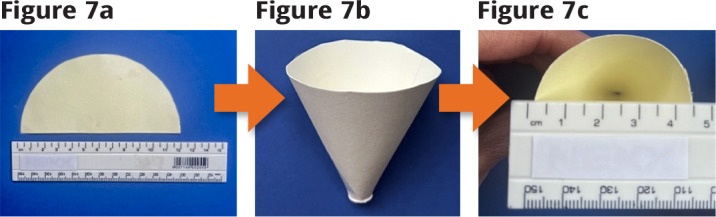


**Figure 8:**
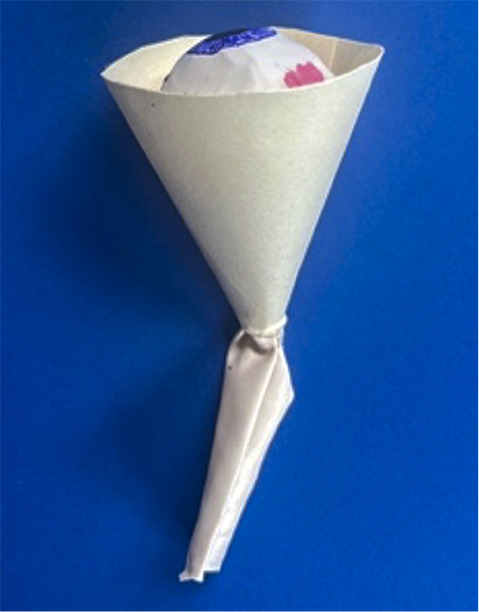


Cut about 5 mm off the tip of the cone to create a small opening for the ‘tail’ of the balloon to pass through. Place the balloon-covered ball into the cone, pulling the tail end through the small opening (Figure 8). The ‘eyeball’ should now fit snugly in the ‘socket’, with a small amount of movement still possible.

**Figure 9:**
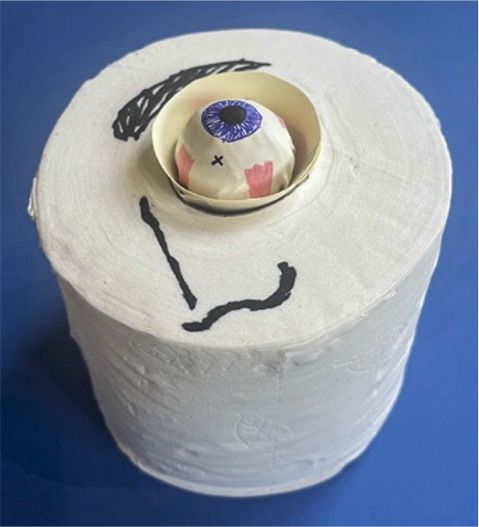


Place the cone into the toilet roll as shown in Figure 9. Draw a ‘nose’ and an ‘eyebrow’ on the toilet roll to serve as an orientation guide.

The cone can be tilted to simulate the patient looking supero-temporally during the procedure. All four quadrants can be used, rotating the cone appropriately so as to always target the inferonasal quadrant.

## How to practice the block

The surgeon stands at the head of the patient model, with the patient looking supero-temporally. Position the eye appropriately.

**When performing the procedure on a patient:** Prepare the conjunctiva by instilling topical anaesthetic drops, instil a drop of povidone iodine 5% solution, and place a speculum.

**Figure 10:**
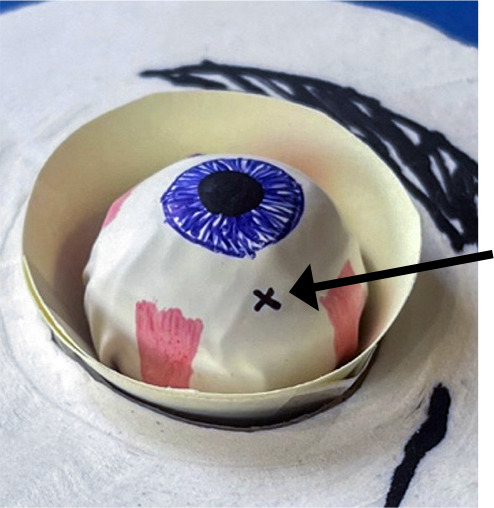


The planned incision, which should be 3–4 mm long, should be 5–8 mm from the limbus in the inferonasal quadrant, midway between the inferior and medial rectus muscles. The ideal location for the incision can be indicated on the balloon using a marker (Figure 10).

Be sure to target posterior to the anatomical insertion of the Tenon's fascia, located 1–2 mm from the limbus, to successfully access the sub-Tenon's space.

**Figure 11:**
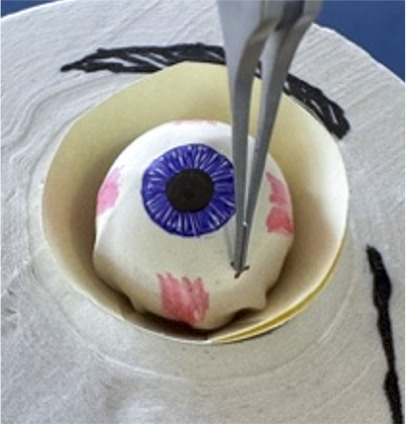


Press down perpendicularly to the sclera (ball) with the tissue forceps in your non-dominant hand and firmly grasp and lift the conjunctiva and tenon's fascia (2 balloon layers) together, creating a small ‘tent’ (Figure 11).

With the scissors in your dominant hand, make a small cut just anterior to the grasping point, with the tip of the curved scissors perpendicular or at 45 degrees to the sclera (ball). Cut through both ‘conjunctiva’ and ‘Tenon's’ (the two balloon layers) together, usually with a single cut, to access bare ‘sclera’ (the ball). This can be confirmed by identifying the lifted coloured balloons held by the forceps.

**Figure 12:**
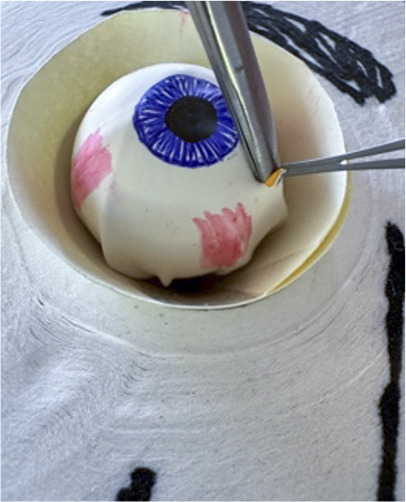

The next step is to create a channel into the sub-Tenon's space (between the Tenon's and the sclera, i.e. between the ball and the first balloon) – this is where the cannula will go. While still grasping the conjunctiva and Tenon's with the forceps, advance the closed scissors 10 mm in a radial direction into the sub-Tenon's space, with the curve of the scissors following the curve of the sclera, allowing the scissors to open slightly (see Figure 12).

**When performing the procedure on a patient:** A small amount of blunt dissection may be required to create a plane between the sclera and the Tenon's layer. However, try not to make the opening or the channel excessively big, as this will enable the anaesthetic to reflux easily out of the space during injection.

**Figure 13:**
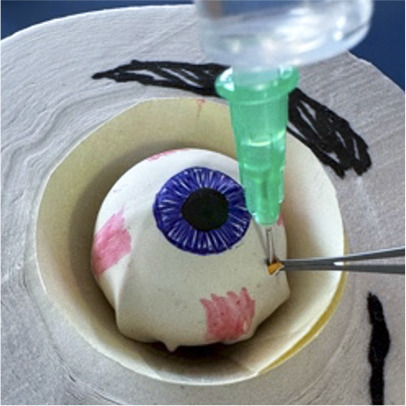


Maintaining the grip with the forceps, gently remove the scissors. Take the syringe with the cannula and advance it into the sub-Tenon's space with the curve following the surface of the sclera, aiming to position the tip against the sclera, just posterior to the equator of the eyeball (Figure 13). This is the ideal injection position.

With the cannula now in place, ‘close’ the opening by bringing the tissues in the forceps against the scleral surface, which may encourage a more posterior flow of the anaesthetic. Slowly inject the desired amount of anaesthetic, usually about 3–4 ml. On the model it is useful to only inject a small amount of water (<1 ml) to prevent wetting the entire setup.

Immediate reflux of the anaesthetic suggests that the space has not been adequately opened, and the scissors are used again to gently extend the space.

**When performing the procedure on a patient:** After the injection, you may place light pressure on the conjunctiva to reduce bleeding.

**CAUTION!** The sub-Tenon's space is intraconal, providing access to the optic nerve, short ciliary vessels, and vortex veins. Even though the cannula is blunt, it is still possible to damage vital tissue if you make aggressive, sweeping, or very posterior movements.

Further information on the finer points of the technique, as well as cautions and possible complications, are described here: bit.ly/3weZ8cL3^2^
